# New Insights into the Role of Sphingolipid Metabolism in Melanoma

**DOI:** 10.3390/cells9091967

**Published:** 2020-08-26

**Authors:** Lorry Carrié, Mathieu Virazels, Carine Dufau, Anne Montfort, Thierry Levade, Bruno Ségui, Nathalie Andrieu-Abadie

**Affiliations:** 1 Centre de Recherches en Cancérologie de Toulouse, Equipe Labellisée Fondation ARC, Université Fédérale de Toulouse Midi-Pyrénées, Université Toulouse III Paul-Sabatier, Inserm 1037, 2 avenue Hubert Curien, CS 53717, 31037 Toulouse CEDEX 1, France; lorry.carrie@inserm.fr (L.C.); mathieu.virazels@inserm.fr (M.V.); carine.dufau@inserm.fr (C.D.); anne.montfort@inserm.fr (A.M.); thierry.levade@inserm.fr (T.L.); bruno.segui@inserm.fr (B.S.); 2 Laboratoire de Biochimie Métabolique, CHU, 31059 Toulouse, France

**Keywords:** cancer, ceramide, gangliosides, immunotherapy, metastasis, phenotype switching, sphingosine 1-phosphate

## Abstract

Cutaneous melanoma is a deadly skin cancer whose aggressiveness is directly linked to its metastatic potency. Despite remarkable breakthroughs in term of treatments with the emergence of targeted therapy and immunotherapy, the prognosis for metastatic patients remains uncertain mainly because of resistances. Better understanding the mechanisms responsible for melanoma progression is therefore essential to uncover new therapeutic targets. Interestingly, the sphingolipid metabolism is dysregulated in melanoma and is associated with melanoma progression and resistance to treatment. This review summarises the impact of the sphingolipid metabolism on melanoma from the initiation to metastatic dissemination with emphasis on melanoma plasticity, immune responses and resistance to treatments.

## 1. Introduction

**Figure 1:**
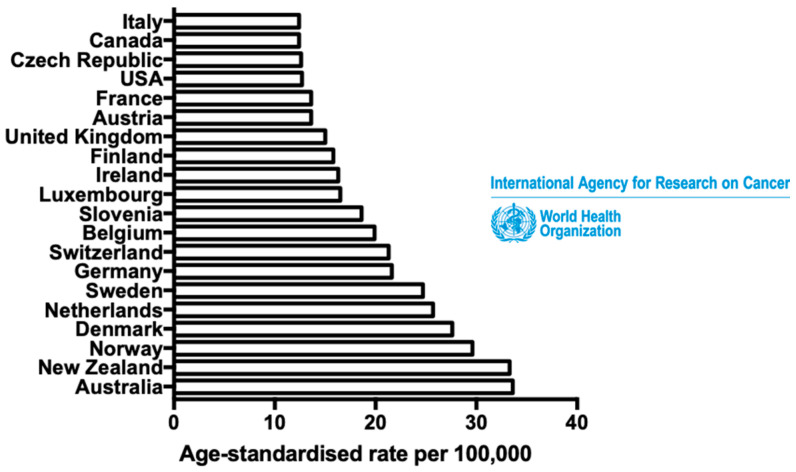
Cutaneous melanoma: the 19th most common cancer worldwide. Estimated age-standardised incidence rates of cutaneous melanoma in the most affected countries in 2018, for both sexes and all ages. Data from the International Agency for Research on Cancer (World Health Organisation).

Cutaneous melanoma is a skin cancer whose incidence is increasing significantly worldwide (Figure 1). Even though melanoma is not frequent, accounting for less than 5% of skin cancers, it can be very aggressive and causes more than 75% of all skin cancer deaths [1] (Figure 1). Despite significant improvement of treatment strategies in the last decade, owing both to the emergence of BRAF- or MEK-targeted therapies and checkpoint blockade immunotherapies (i.e., anti-cytotoxic T-lymphocyte-associated antigen-4 (CTLA4) and anti-programmed cell death-1 (PD-1)), the prognosis for patients with metastatic melanoma remains uncertain, predominantly due to treatment failures and recurrences [2]. Fortunately, melanoma is usually curable by excisional surgery if detected at an early stage, with a high five-year survival rate [3,4]. Thus, a better understanding of melanoma progression processes, before dissemination, is a major public health issue in order to discern new therapeutic targets.

**Figure 2:**
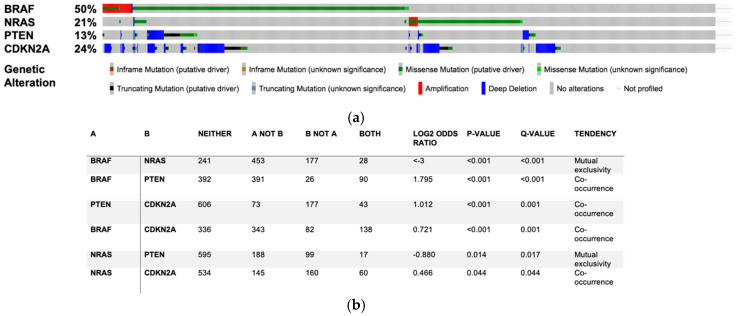
*BRAF*, *NRAS*, *PTEN* and *CDKN2A* are the most frequently mutated genes in cutaneous melanoma. Mutation rate, genetic alteration (**a**) and mutual exclusivity (**b**) for *BRAF*, *NRAS*, *PTEN* and *CDKN2A* mutations observed in 1635 samples from 1584 patients included in 12 studies analysed on cBioportal for cancer genomics (https://www.cbioportal.org).

Melanoma arises from melanocytes, i.e., melanin-producing neural crest-derived cells, which are located at the junction between the epidermis and the dermis [5]. The initial stage of melanomagenesis corresponds to a radial-growth phase (RGP), in which melanoma cells invade laterally but stay confined into the epidermis. This stage is followed by a vertical-growth phase (VGP), in which melanoma cells invade the dermis and are able to reach blood vessels. Then, an extravasation stage, corresponding to the release of melanoma cells from the blood circulation into new tissues, leads to the formation of metastatic niches [6,7,8]. Melanomagenesis requires at least two key events. The first is activating mutations in oncogenes such as *BRAF* or *NRAS*. BRAF mutations are found in 50% of melanoma patients (Figure 2) and the V600E mutation accounts for approximately 75% of all BRAF mutations detected in cutaneous melanoma [9]. Conversely, the most common NRAS mutations, i.e., Q61R and Q61K, affect about 20% of melanoma patients [10]. Since BRAF and NRAS mutations are mutually exclusives, these driver mutations lead to constitutive activation of the mitogen-activated protein kinase (MAPK) pathway and aberrant cancer cell proliferation in approximately 70% of patients (Figure 2) [11]. The second event is illustrated by the loss of expression of key tumour suppressor genes such as *PTEN* or cell cycle checkpoint regulators such as *CDKN2A,* which occur in 13% and 24% of patients, respectively (Figure 2). These genetic changes can bypass oncogene-induced senescence (OIS) processes and cause the immortalisation of tumour cells [4,7,12] (Figure 2).

Metabolic reprogramming is also crucial for melanomagenesis. Indeed, a shift from mitochondrial oxidative phosphorylation to cytoplasmic anaerobic glycolysis, known as Warburg effect, is required for metastatic dissemination [13]. The present review focuses on alterations in the metabolism of sphingolipids (SL). Interestingly, several key enzymes of the glycolytic pathway can be severely affected by changes in SL metabolism in melanoma. For instance, C16-ceramide, which is the major long-chain ceramide in melanocytes and melanoma cells, impairs pyruvate kinase, hexokinase and LDH activities, consequently altering cellular glycolysis and inhibiting melanoma progression [14].

How modulations of the SL metabolism affect dermatologic diseases have long been studied [15] and accumulating evidence demonstrates the presence of alterations in the ceramide metabolism in melanoma cells. This article aims at providing a comprehensive overview of the effects dysregulations of the SL metabolism have on melanomagenesis, melanoma progression, immunity and resistance to treatment, especially linked to the phenotype switching.

## 2. Alterations of Sphingolipid Metabolism in Melanoma

**Figure 3:**
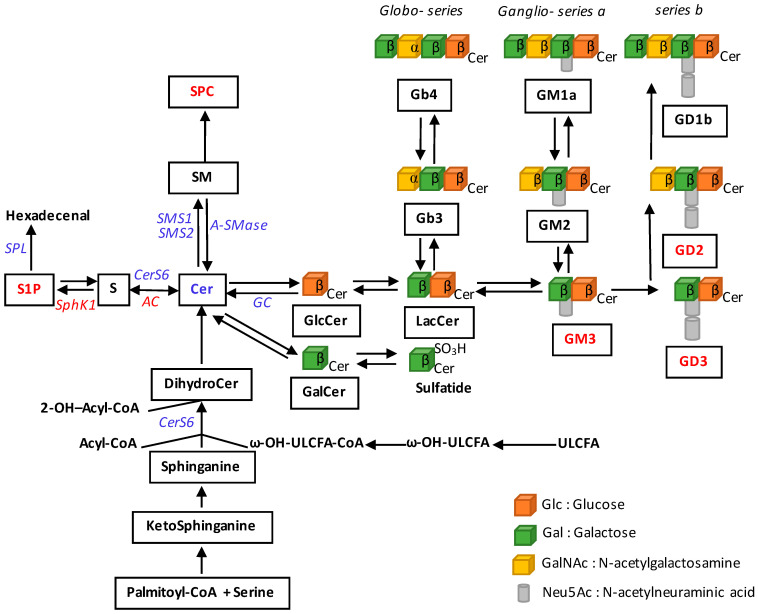
Multiple dysregulations of sphingolipid metabolism in melanoma. SL metabolites or SL-metabolising enzymes whose levels or expression are altered in melanoma, are mentioned. Decreases are indicated in blue and increases in red. AC, acid ceramidase; Cer, ceramide; CERS, ceramide synthase; CoA, coenzyme A; DihydroCer, dihydroceramide; GalCer, galactosylceramide; GC, glucosylceramidase; GlcCer, glucosylceramide; LacCer, lactosylceramide; S, sphingosine; S1P, sphingosine 1-phosphate; SM, sphingomyelin; SMases, sphingomyelinases; SMS, sphingomyelin synthase; SPC, sphingosylphosphorylcholine; SphK, sphingosine kinase; SPL, S1P lyase; ULCFA, ultralong chain fatty acids.

The main dysregulation affecting the SL metabolism in melanoma cells is a trend toward a reduction of ceramide, which promotes cell death (for review, see [16]). This is associated with changes in the expression and/or activity of a number of enzymes as well as the accumulation of tumour-promoting metabolites, which include sphingosine 1-phosphate (S1P) and gangliosides (Figure 3).

The impact of SL metabolism dysregulations in melanoma cell lines and/or patients is summarised in Table 1. For instance, a low expression of the ceramide synthase 6 (CerS6) in melanoma cells is related to malignant behaviours as demonstrated in WM35, WM451 and SKMEL28 human melanoma cell lines [17]. In addition, acid ceramidase (AC), encoded by the *ASAH1* gene, which hydrolyses ceramide into sphingosine, is expressed at high levels in melanocytes and proliferative melanoma cells, as observed in vitro as well as in biopsies from patients with stage II melanoma [18]. *ASAH1* expression was: (i) higher in human melanoma cell lines exhibiting a proliferative phenotype as compared to invasive ones; and (ii) reduced at the invasive front on tumour specimens from melanoma patients [19]. Sphingosine kinase 1 (SphK1), which phosphorylates sphingosine to produce sphingosine-1-phosphate (S1P), also shows increased expression and/or activity in melanoma cells compared to melanocytes, not only in human and murine cell lines [20,21] but also in human biopsies [22]. Collectively, these findings suggest a shift of the S1P-ceramide balance towards S1P production in melanoma cells. In accordance, the expression of *SGPL1* gene, encoding for S1P lyase (SPL), is downregulated in melanoma cell lines when compared to adult or juvenile melanocytes, suggesting that *SGPL1* might be downregulated during melanomagenesis [23].

Ceramide can also be converted into more complex SL, such as gangliosides. For instance, mono- and disialoganglioside levels are very high in human melanoma cells and tissues, especially GD3 [24]. Interestingly, levels of this latter ganglioside were correlated to the expression of the c-Yes tyrosine kinase whose activity is known to increase in melanoma cells as compared to melanocytes [25] and whose inhibition reduced the malignant potential of GD3^+^ melanoma cells only [26].

Alterations of the ceramide metabolism also include changes in the expression of sphingomyelin synthase 1 (SMS1), which is encoded by the *SGMS1* gene and catalyses the transformation of ceramide into sphingomyelin (SM) [27,28]. *SGMS1* is expressed at low levels in most of the human melanoma biopsies and low *SGMS1* expression is associated with a worse prognosis in metastatic melanoma patients. Of interest, a weak expression of *SMS1* was shown not to be associated with an intracellular accumulation of ceramide, most likely due to its conversion into glucosylceramide (GlcCer) through GlcCer synthase (GCS). Consequently, 6 out of 10 human melanoma cell lines exhibited higher levels of GlcCer than SM [29]. Moreover, SM can also be transformed into sphingosylphosphorylcholine (SPC) by a yet uncharacterised SM deacylase and SPC has been shown to stimulate regulators of melanomagenesis such as extracellular signal-regulated kinases (ERK), microphthalmia-associated transcription factor (MITF) and Akt/mTOR [30,31,32,33].

Finally, the expression of acid sphingomyelinase (A-SMase), which hydrolyses SM into ceramide, has been shown to be higher in benign nevi than in primary melanomas, and further reduced in the lymph-node metastases [34]. Moreover, a lower expression/activity of A-SMase was observed in hyper-pigmented murine and human melanomas as compared to the hypo-pigmented ones, suggesting an inverse correlation between A-SMase expression/activity and melanin content. In accordance, exogenous C2-ceramide decreased melanin content in melanocytes [35].

## 3. Role of the Sphingolipid Metabolism in Melanomagenesis

### 3.1. Do SL Metabolism Alterations Increase the Risk to Develop Melanoma?

A genome-wide association study has identified the 1q21.3 chromosomal region, containing LASS2 gene that encodes ceramide synthase 2 (CerS2), as a locus predisposing to cutaneous melanoma [36]. These observations suggest the involvement of some genetic determinants of the ceramide metabolism in melanoma predisposition. Interestingly, a defect in glucosylceramidase 1 (GBA1) resulting in Gaucher inborn disorder (GD), owing to a defect in GlcCer hydrolysis into ceramide, is associated with an increased risk of malignancies, including melanoma [37,38]. Indeed, accumulation of GlcCer as well as glucosylsphingosine, which arises from the cleavage of excess GlcCer by AC [39,40], occurs in macrophages and could severely alter the immune and inflammatory responses. This could create a favourable microenvironment to promote melanomagenesis (for review see [38]).

Another hypothetical link between glucosylceramidase (GC) and melanoma development is autophagy, which can be either cell protective, promoting their survival, or lethal, via induction of a programmed-cell death mechanism [41,42]. Defective autophagy has been reported in models of GC or saposin C deficiency [43]. Accumulation of GlcCer was associated to autophagy dysfunction in a drosophila model of GD that lacked the two fly GBA1 orthologues [44]. In accordance, GlcCer accumulation was also associated with autophagy impairment and defective autophagosome-lysosome fusion, resulting in autophagosome accumulation in induced pluripotent stem cells (iPSCs) derived from patients with GD [45]. Moreover, an hyperactivation of the autophagic inhibitor mTOR and a downregulation of the master regulator of lysosome function TFEB were reported in human neuroglioma cells treated with the GC inhibitor conduritol B epoxide [46]. Numerous studies have shown that impaired autophagy can favour melanoma development. Indeed, the activation of mTOR was associated with poor prognosis in melanoma patients [47]. In addition, ERK-induced TFEB phosphorylation impaired expression of autophagy-lysosome target genes in BRAF-mutated melanoma, which elicited the formation of TGF-β-dependent metastases [48].

All these findings indicate a possible link between GC deficiency and melanomagenesis, that may result from an altered immune response or disturbed autophagy. The underlying mechanisms remain, however, to be determined.

### 3.2. Sphingolipid Metabolism Modulates Melanoma Cell Proliferation and Survival

Transformation of normal melanocytes into melanoma cells is mediated by the activation of growth stimulatory pathways, typically leading to cellular proliferation as well as the inactivation of apoptotic and tumour suppressor pathways. The RAS-RAF-MEK-ERK pathway is one of the most important signalling pathways involved in melanoma cell growth and survival [49,50]. A constitutive activation of BRAF, mostly due to the substitution of valine by glutamic acid at position 600 (also known as V600E), affects about 50% of melanoma patients [9,51,52,53] (Figure 2). Therapies targeting the BRAF V600E mutation help advanced melanoma patients live longer [54,55]. Moreover, co-administration of BRAF (vemurafenib) and MEK (cobimetinib) inhibitors improves the progression-free survival [56] and extends the five-year overall survival by ~40% [57]. Unfortunately, most patients, including those who experience an initial tumour regression, exhibit disease progression within 6–8 months following the initiation of targeted therapy [58].

Multiple lines of evidence indicate that some SL-metabolising enzymes regulate melanoma cell proliferation and survival. First, SphK1 expression and activity are induced by ERK1/2 and AKT in numerous mammalian cells [59,60,61], including melanoma cells [20,21]. Moreover, SphK1 knockdown by siRNA decreased anchorage-dependent and -independent growth of human melanoma cells [20]. Similarly, targeting SphK1 using shRNA in B16F10 [62] or Yumm 1.7 [22] murine melanoma cells reduced tumour growth in syngeneic mice. Accordingly, the SphK1 inhibitor SKI-I, which increases the intracellular ceramide levels and decreases S1P levels in melanoma cells, resulted in a cell cycle arrest between G2/M and S phases as well as increased apoptotic cell death, caspase-3 activation and nuclear accumulation of cleaved PARP [20]. The intraperitoneal administration of SKI-I in mice harbouring melanoma also decreased tumour growth [20,22]. Consistently, the growth of B16F10 tumours is impaired in SphK1^−/−^ mice as compared to wild-type animals [21].

S1P, which is mainly produced by SphK1 in melanoma cells, conveys oncogenic signals as an intracellular second messenger via a ligation of a family of G-protein coupled receptors (S1P1-5) expressed both on the malignant and their neighbouring cells [63]. We recently demonstrated that the melanoma cell-autonomous survival in response to the BRAF inhibitor vemurafenib is mediated by S1P1 and S1P3 [64].

Moreover, AC, which is expressed at high levels in proliferative melanoma cells, may also contribute to melanoma cell proliferation and survival. Indeed, AC inhibition by siRNA dramatically reduced the number of 501mel melanoma cells, as shown using short-term cell growth and colony formation assays [19]. Similarly, CRISPR/Cas9-mediated AC ablation in A375 melanoma cells blocked G1/S cell cycle progression, promoted senescence and apoptosis, resulting in reduced cell growth. These cells were unable to form spheroids and showed a lower replication rate as well as a decreased in their invasive capacity compared to controls. Mechanistically, AC ablation resulted in the accumulation of the saturated C14-, C16- and C18-ceramides and is accompanied by the down-regulation of MYC, CDK1, CHK1 and AKT [65]. In accordance, the inhibition of AC activity with a chemically stable AC inhibitor, named compound ARN14988, sensitised proliferative melanoma cells to the cytotoxic actions of various anti-tumour agents [18]. In line with these observations, we previously reported that the cytotoxic action of dacarbazine was accompanied with AC proteolysis in human melanoma cells [66]. Of interest, confocal immunofluorescence analyses revealed the nuclear localisation of AC in normal melanocytes, a phenomenon not observed in melanoma cells, suggesting that AC could activate proliferation pathways only in tumour cells [18].

By reducing ceramide levels, AC, in concert with SphK1, favours melanoma cell proliferation. This was confirmed using short-chain C2-ceramide, which was reported to inhibit AKT and ERK activation as well as proliferation in Malme-3M melanoma cells [67]. In addition, the GCS inhibitor PDMP, which increases intracellular C16-ceramide levels, inhibited cell proliferation, migration and invasion of WM35 and WM451 human melanoma cells. The effect of PDMP was associated with the inhibition of key enzymes from the glycolysis pathway including the pyruvate kinase, hexokinase and lactic acid dehydrogenase. Strikingly, the treatment of melanoma cells with exogenous C16-ceramide neither altered melanoma cell growth nor migration and invasion. In contrast, exogenous C16-ceramide was shown to promote glycolysis. This opposite effect could be explained by the reduction of endogenous C16-ceramide levels, which was induced by exogenous C16-ceramide treatment [14].

Finally, GCS, which catalyses the first committed step in the synthesis of most glycosphingolipids, i.e., the transfer of glucose to ceramide to form GlcCer, is also able to control tumorigenic capability of melanoma cells. Indeed, antisense oligonucleotide targeting the Ugcg gene, encoding GCS, reduced tumorigenicity of MEB4 murine melanoma cells [68]. Similarly, the inhibitor of GCS, OGT2378, inhibited MEB4 melanoma tumour growth in a syngeneic, orthotopic murine model [69]. In agreement with these findings, we previously showed that overexpression of GBA2 in melanoma cells, an enzyme able to degrade GlcCer into ceramide, reduced tumour cell growth both in vitro and in vivo by triggering ER stress-induced apoptosis [70]. Of note, GBA2 gene is downregulated in melanoma cells as compared to melanocytes [70]. Altogether, these observations demonstrate that the transformation of ceramide into GlcCer facilitates melanoma cell proliferation.

Interestingly, sialic acid-containing glycosphingolipids, i.e., gangliosides, can also regulate melanoma cell proliferation. First, treatment of SKMEL-28 melanoma cells with the anti-GD3 antibody R24 reduced their growth in vitro and decreased their tumorigenicity when injected in immunodeficient mice [71]. Second, Furukawa et al. demonstrated that GD3 increased the proliferation of GD3 synthase-overexpressing melanoma cells [72]. In these settings, GD3 mediated the convergence of several pro-tumoral signals, including those induced by hepatocyte growth factor (HGF) and the receptor tyrosine kinase c-MET, notably promoting cell proliferation [73].

Altogether, these data illustrate that SL metabolism alterations, which redirect ceramide metabolism towards S1P or GD3 production, can promote melanoma cell proliferation and survival in response to drugs.

## 4. Role of the Sphingolipid Metabolism in Melanoma Progression

### 4.1. SL Metabolism Regulates Melanoma Cell Adhesion

Cell junctions, which are crucial for the communication between neighbouring cells and with the extracellular environment, can be divided into three major classes: anchoring junctions (including adherens junctions, desmosomes, hemidesmosomes and focal adhesions), tight junctions and gap junctions. In the epidermis, cadherins are the major adhesion molecules, especially involved in the composition of desmosomes and adherens junctions [74], whereas integrins are the major component of hemidesmosomes [75]. Among cadherins, E-cadherin mediates the adhesion between melanocytes and keratinocytes allowing keratinocytes to control cell growth and dendricity of melanocytes [76]. E-cadherin expression is lost in melanoma cells during the first steps of tumour progression [77]. Interestingly, when E-cadherin expression is restored, keratinocytes recover control of melanoma cells thus preventing tumour progression [78].

Here, we review studies documenting the role of the SL metabolism in the control of the expression of adhesion molecules as well as melanoma cell adhesion capacity. Previous reports have shown that E-cadherin loss was observed in SphK1-overexpressing cancer cells [79,80]. S1P-induced E-cadherin downregulation could be mediated by S1P2 and S1P3, as shown in alveolar epithelial cells [81] and lung fibroblasts [82]. This phenomenon could be indirect in melanoma cells as the SphK1/S1P pathway is able to stimulate TGF-β1 production [62], which may trans-activate S1P2 and S1P3 [82]. Interestingly, overexpression of S1P2, but not S1P1, in B16F10 melanoma cells resulted in the inhibition of the small GTPase Rac activity as well as tumour progression in mice [83]. Importantly, Rac is crucial to create E-cadherin-dependent cell-cell contacts [84].

Moreover, downregulation of AC in melanoma cells induced E-cadherin loss and, inversely, increased expression of the epithelial-mesenchymal transition (EMT)-associated protein TWIST1, which is in accordance with a more aggressive phenotype [19].

Finally, GD3 was shown to favour the recruitment of integrins through glycolipid-enriched microdomains in GD3 synthase-overexpressing melanoma cells. Under these conditions, melanoma cell adhesion to the extracellular matrix (ECM) was increased [85]. Similarly, Ohmi et al. demonstrated that cell adhesion increased in GD2 synthase-overexpressing melanoma cells as compared control cells [86].

Thus, SL alterations appear to impact on melanoma cell adhesion, particularly through E-cadherin loss, which promotes melanoma progression.

### 4.2. SL Metabolism as a Determinant of Melanoma Plasticity

To colonise distant organs, tumour cells need, besides losing their cell junctions, to acquire invasive capacities. In skin cancers, EMT plays a key role in this process. This fundamental mechanism allows epithelial cells to gain mesenchymal features, increasing their migration and invasion abilities (for review, see [87]). However, unlike other skin cancers, melanoma does not arise from epithelial cells but from neural crest-derived melanocytes. For this reason, EMT stricto sensu cannot be considered in melanoma progression. Nevertheless, an EMT-like phenomenon has been described, in which melanoma cells can dynamically and reversibly switch between a proliferative and an invasive state; this is known as “phenotype switching”. Indeed, the microarray analysis of DNA from different human melanoma cell lines allowed Hoek et al. to determine a transcriptional signature representative of metastatic cell behaviour. The authors indeed demonstrated that MITF is one crucial actor in this switch, particularly in maintaining the proliferative state [88].

MITF represents a melanocytic lineage-specific transcription factor that regulates melanocyte differentiation, function and survival as well as melanoma progression [89]. MITF regulates pigment cell-specific transcription of genes encoding melanogenic enzymes such as TYR, DCT and TYRP1, as well as proteins involved in melanosome formation and maturation such as Melan-A, Premelanosome Protein and G Protein-Coupled Receptor 143. As a matter of fact, MITF expression and activity are modulated by a range of activators and suppressors operating at transcriptional, post-transcriptional and post-translational levels (for review, see [90]). MITF function has been tightly connected to melanoma cell plasticity. It is now well accepted that melanoma cells expressing moderate to high levels of MITF proliferate rapidly and are poorly invasive, whereas melanoma cells characterised by low MITF levels grow more slowly and are more invasive. Thereby, low levels of MITF correlate with a worse prognosis for melanoma patients [19,89,91].

Interestingly, numerous studies showed that the SL metabolism regulates MITF expression. Firstly, A-SMase expression has been shown to induce ERK-mediated MITF degradation by the proteasome. Therefore, the loss of A-SMase observed during melanoma progression accounts for the upregulation of MITF as well as for some of its downstream targets CDK2, Bcl-2 and c-MET [34]. Secondly, AC ablation by the CRISPR-Cas9 technology, which is associated with the accumulation of long-chain saturated ceramides, led to a strong downregulation of MITF expression in human A375 melanoma cells, reducing their ability to form cancer-initiating cells and to undergo self-renewal [65]. Furthermore, exogenous addition of C2-ceramide was shown to reduce MITF expression in human melanocytes [35]. Reciprocally, we demonstrated that MITF expression increased in AC-overexpressing melanoma cells. However, at variance with Lai et al., we observed that melanoma cells expressing AC at high levels displayed a proliferative phenotype as compared to cells with low expression of AC that exhibited high mobility and gain of mesenchymal features [19]. Moreover, using a ChIP-Seq database, we identified AC as a new target of MITF, demonstrating that MITF and AC are part of a positive feedback loop.

SL metabolism could also modulate MITF levels by acting on signalling pathways known to regulate its expression in melanoma cells. For instance, canonical Wnt signalling through the Wnt/β-catenin pathway is a critical activator of MITF expression in melanoma cells [92], and deactivation correlates with a higher metastatic potential [88]. Interestingly, exogenous sphingosine has been shown to reduce nuclear and cytosolic β-catenin expression in SW480 and T84 colon cancer cells [93]. In accordance, pharmacological inhibition of SphK1 with SKI-II was associated to a decreased β-catenin expression in human hepatoma carcinoma [94]. As expected, FTY720, a sphingosine analogue known to inhibit SphK1 [95], led to the reduction of β-catenin as well as MITF expression in melanocytes [96].

Moreover, a switch in EMT-associated transcription factors (EMT-TFs) occurs in melanoma and drives tumour progression. This dynamic network includes Snail, Zeb and Twist families, which are major repressors of epithelial genes, and, conversely, major activators of mesenchymal genes (for review, see [87]). In particular, a reduced expression of ZEB2 and SNAIL2 in favour of an increased expression in ZEB1 and TWIST1 was linked to E-cadherin loss, increased invasion properties and poor clinical outcomes in human melanoma [97]. The EMT-TFs switch was associated with a reduction of MITF expression. Indeed, ZEB1 and TWIST1 have been shown to downregulate MITF whereas SNAIL2 or ZEB2 induce MITF expression, demonstrating that these EMT-TFs act as key players in melanoma phenotype switching.

Recent studies identified a strong connection between SL metabolism and EMT-TFs. Indeed, reduced expression of CerS6, which decreased the levels of intracellular C16-ceramide, was associated with an increased expression of SNAIL2 in SW480 colon cancer cells [98]. Hence, by controlling the expression of EMT-TFs or by altering plasma membrane fluidity, C16-ceramide could affect cancer cell motility [99]. Another SL-metabolising enzyme could also account for the effects of ceramide on EMT-TFs. Indeed, SMS2, which produces SM from ceramide, seems to stimulate the expression of mesenchymal markers and enhance migration and invasion of MCF-7 and MDA-MB-231 breast cancer cell lines. Interestingly, SMS2 expression was higher in metastatic breast cancer than in non-metastatic tumours. Mechanistically, SMS2 was shown to activate the canonical TGFβ/SMAD signalling pathway leading to the expression of its downstream target Snail [100]. In addition, previous studies have demonstrated that some ganglioside-metabolising enzymes are connected with EMT-FTs and gangliosides play a critical role in EMT [101]. For instance, ZEB1 was reported to be a direct regulator of the GM3 synthase gene (St3gal5) in mammary epithelial NM18 cells. ZEB1 also impaired the expression of miR-200a, a microRNA targeting the 3ʹUTR GM3 synthase mRNA. Knockdown of GM3 synthase partly mimicked the effects of ZEB1 inhibition, leading to increased expression of cell junction components such as E-cadherin as well as intercellular adhesion [102]. Moreover, overexpressing TWIST or SNAIL1 in transformed human mammary epithelial cells enhanced the expression of GD3 synthase [103]. GD3 synthase knockdown reduced breast cancer stem cell-associated properties and completely abrogated tumour formation in vivo. In accordance, other studies have shown that ceramide glycosylation by GCS was enhanced in breast [104] and colon [105] cancer stem cells and GCS inhibition significantly decreased the expression of ZEB1 and β-catenin [105]. Whether GCS, a ganglioside-metabolising enzyme, CerS6 or SMS2 modulates EMT-TFs in melanoma cells remains to be evaluated.

Furthermore, numerous studies have shown that TGF-β is a strong promoter of EMT in many tumours [106,107], including melanoma [88]. TGF-β signalling inhibits MITF expression through PAX3 repression and GLI2 activation. Many studies reported that the SphK1/S1P and the TGF-β signalling are interconnected. Indeed, through its binding to S1P receptors, S1P was shown to promote TGF-β receptor trans-activation leading to Smad phosphorylation and cell migration [108,109,110]. Additionally, in different cancers including melanoma, S1P was reported to increase TGF-β expression and secretion [62,111,112]. Inversely, TGF-β was able to increase SphK expression and activity, which were essential to control the effects of TGF-β on extracellular matrix remodelling, cell migration and invasion [113,114].

Finally, TEAD transcription factors (TEADs) were identified as key regulators of the invasive state in melanoma [115]. TEADs need coactivators such as YAP and TAZ, which are known effectors of the Hippo pathway. This pathway has been shown to modulate Wnt and TGF-β signalling and confer pro-invasive properties in melanoma [116]. Strikingly, multiple studies have identified S1P as an activator of YAP through S1P2 signalling [117,118,119]. Similarly, a recent study demonstrated that inhibition of SphK1 using the PF-543 inhibitor could inhibit TGFβ-induced activation of YAP [120]. As anticipated from its close structural similarity with S1P, SPC also regulated the Hippo pathway via S1P2, in a rather unclear manner as it could both inhibit and activate YAP [121].

To summarise, tight connections have been reported between SL metabolism and key players of the phenotype switching in melanoma including transcription factors such as MITF, EMT-TFs, TEADs and fundamental signalling pathways such as Wnt, TGF-β and Hippo. These observations further highlight the importance of the SL metabolism in melanoma progression.

### 4.3. SL Metabolism as a Major Regulator of Melanoma Aggressiveness

As discussed above, melanoma aggressiveness depends on the balance between its proliferative potential and migratory/invasive properties. S1P was reported either to activate or to inhibit melanoma cell migration depending on S1P receptor subtypes. Indeed, whereas S1P inhibited cell migration, with the concomitant inhibition of Rac and stimulation of RhoA, in S1P2-expressing B16F10 cells, it stimulated cell migration of S1P1-overexpressing cells, demonstrating a receptor subtype-specific action of S1P on melanoma cells [83]. The inhibitory effects of S1P were reversed by the S1P2-selective antagonist JTE013, which stimulated Rac and migration of B16F10 cells overexpressing either S1P1 or S1P3 [122]. Similar results were obtained in B16F10 cells treated with SPC instead of S1P [122].

In addition, AC overexpression in melanoma cells decreased tumour cell motility, whereas AC silencing had the opposite effect, as was observed for MITF [19]. We recently demonstrated that low AC expression was associated to increased FAK phosphorylation and relocation at focal adhesions instead of cytoplasm. This phenomenon led to increased expression of integrin β5 (ITGβ5) and integrin αV (ITGαV), which play a critical roles in the migratory and invasive capacity of cancer cells. As a result, the melanoma invasive behaviour induced by AC inhibition was reduced using an ITGαVβ5 blocking antibody [19].

Another study reported that lung metastases were reduced in A-SMase-deficient mice injected with B16-F10 melanoma cells. Treating B16F10 cells with exogenous A-SMase or C16-ceramide before inoculation restored lung metastatic lesions in A-SMase-deficient mice. Mechanistically, melanoma cells were shown to activate A-SMase in platelets, leading to ceramide production, which favoured the clustering and activation of α5β1 integrins at the surface of melanoma cells and therefore tumour cell adhesion in the lungs [123]. As the expression of melanoma A-SMase negatively correlates with tumour aggressiveness [34], one could speculate that A-SMase expression acts as a key factor that controls melanoma cell invasion and adhesion into the metastatic niches.

Gangliosides also likely contribute to melanoma cell dissemination. As a matter of fact, the level of GM3, which has been described as one of the major gangliosides in melanoma [124], increased in murine metastatic melanoma [125], suggesting a role for GM3 in tumour aggressiveness. Indeed, the addition of GM3 to B16LuF1 melanoma cells, i.e., B16-melanoma cells of lower metastatic potential to lungs, increased their dissemination capacity once injected in mice [126]. Liu et al. also described that de-N-acetyl GM3 (d-GM3), a derivative of ganglioside GM3, was mainly found in metastatic melanomas but not in benign nevi or most primary melanomas. d-GM3 expressing melanoma cells possess increased migratory and invasive capacities as compared to melanoma cells lacking d-GM3. Mechanistically, d-GM3 stimulated MMP-2 expression via the urokinase-like plasminogen activator (uPA) receptor [127].

Some studies indicated that GD3 also stimulates melanoma cell invasion. Indeed, human melanoma GD3-positive cells showed a markedly increased cell invasion potential as compared to GD3-negative cells. The invasive activity induced by GD3 was shown to be mediated by p130Cas or paxillin, two components of the focal adhesion cytoskeleton [72]. More recently, Ohmi et al. compared the effect of GD3 and GD2 on melanoma progression. Using GD3-^high^ or GD2-^high^ melanoma cells, obtained by overexpressing the respective glycosyltransferases involved in their production, they demonstrated that GD2 enhanced the adhesion properties of melanoma cells, while GD3 stimulated their invasive capacities. These findings led the authors to propose that GD2 would rather act at the primary and metastatic sites in order to promote cell proliferation and dissemination, while GD3 would favour melanoma cell invasion in order to reach a metastatic niche [86].

Finally, emerging literature indicates that tumour exosomes actively participate in tumour invasiveness and favour the formation of pre-metastatic niches in various types of cancer, including melanoma [128,129,130]. Exosomes are small extracellular vesicles (EVs) that originate from the fusion of multivesicular bodies with the plasma membrane and convey their cargo towards target cells. They carry transmembrane and cytosolic proteins, DNA and small RNAs [131]. In vitro, melanoma-derived exosomes were shown to promote the EMT-like processes in primary melanocytes. This effect occurred in an autocrine/paracrine fashion and was mediated by the microRNA Let-7i [132]. In mice, B16-F10-derived exosomes demonstrated preferential homing to lymph nodes and facilitated the seeding of intravenously injected parental cells [133]. In particular, Peinado et al. showed that, through the receptor c-MET, B16-F10-derived exosomes can educate bone marrow-derived cells, promoting angiogenesis, vascular leakiness, the growth of primary tumours and metastasis [128].

It is well known that ceramide, generated by the neutral SMase2 (nSMase2) on the cytosolic leaflet of endosomal membranes, is involved in the budding of exosomes [134]. Mechanistically, the cone-shaped structure of ceramide could induce spontaneous negative curvature by creating an area difference between the membrane leaflets [135]. Moreover, a decrease in the activity of nSMase2 induced by the GW4869 compound, resulted in the reduced release of exosomal miRNAs [136]. By controlling exosomal miRNA secretion, nSMase2 is able to promote angiogenesis as well as metastasis [137]. Furthermore, Kajimoto et al. also showed that S1P, produced by SphK2 but not Sphk1, can regulate the cargo content in exosomes [138,139] probably through the Gβγ subunit of Gi proteins coupled with S1P1 [140]. Whether nSMase2- and/or SphK2-dependent exosome formation modulates melanoma progression remains to be investigated.

All these findings clearly establish a close relationship between SL metabolism and melanoma invasion and suggest that SL metabolism could be therapeutically targeted in order to improve the outcome of melanoma patients.

## 5. Role of SL Metabolism in the Immune Response to Melanoma

Melanoma cells harbour an aberrant antigenic profile, which allows for an anti-tumour immune response [141]. Despite their high immunogenicity, melanoma cells eventually evade the immune system, grow and metastasise [142,143]. A growing body of evidence in the literature indicates that SLs regulate various immune processes. Thus, deciphering the role of SL metabolism in melanoma immune escape is of great clinical interest.

### 5.1. S1P in Lymphocyte Traffic and Differentiation

Lymphocytes sense S1P concentration via S1P1 [144,145] allowing their egress from the thymus and lymph nodes to peripheral tissues [146]. S1P1 expression is modulated cyclically during lymphocyte traffic, depending on the local S1P concentration: it is downregulated in the blood, upregulated in secondary lymphoid organs (SLO) and downregulated again in the lymph [147]. CD69, an early activation marker on lymphocyte surface, induces S1P1 internalisation and degradation [148], sequestering lymphocytes in SLO [149] and peripheral tissues [150]. S1P1 downregulation is necessary to establish a long-term memory in the skin [151,152] and CD69 is one of the markers (with CD103) for tissue-resident memory cells (TRM) [153], which play a critical role in melanoma immunosurveillance [154,155,156].

Drouillard et al. proposed that the S1P1/S1P2 ratio dictates the migration of T cells, as S1P2 inhibited the chemo-attraction of peripheral T cells [157]. Sic et al. reported that human B cells also migrate towards S1P in an S1P1-dependent manner that is inhibited by CD69 expression [158]. Interestingly, egress of natural killer (NK) cells from the bone marrow and SLO is mediated by the expression of S1P5 [159,160], which is regulated by T-box transcription factor TBX21 [161]. Increased S1P5 expression as well as downregulation of CXCR4 during NK differentiation is necessary for their egress from the bone marrow [162].

SphK activity and S1P1 expression were shown to mediate differentiation of CD4^+^ T cells to Th1 cells and inhibit induced Treg (iTreg) generation [163]. In accordance, in T cell-specific S1p1-transgenic mice, S1P1 oriented the differentiation of CD4^+^ towards the Th1 lineage when antigen-activated. Moreover, S1P1 overexpression impaired the maintenance of Foxp3 expression in naïve TGF-β-treated CD4^+^ T cells. The differentiation of naïve CD4^+^ T cells towards Th1 or iTreg appeared to be reciprocal, driven by the S1P1-mTOR axis, and dependent on the SphK activity as demonstrated with the SphK inhibitors N,N,-dimethylsphingosine (DMS) and SKI. Similarly, CD4^+^ T cells deficient for Sphk1 showed a lesser Foxp3 expression when cultured with IL-2 and TGF-β [164].

### 5.2. S1P Impairs the Immune Response in Melanoma

We reported that melanoma SphK1 plays a key role in the recruitment and phenotypic switch of TAM notably promoting their commitment to a pro-tumoral M2-like phenotype [62]. Moreover, we recently showed that high SphK1 expression in melanoma cells was associated with shorter progression-free and overall survivals in melanoma patients treated with anti-PD-1-based immunotherapy. In mice, SphK1 knockdown in melanoma tumours potently reduced the production of a number of immunosuppressive cytokines including TGF-β [22,62], limiting Treg tumour infiltration. Under these conditions, the response of melanoma cells to anti-PD-1 or anti-CTLA-4-based immunotherapy highly increased [22]. Interestingly, Chakraborty et al. also reported that tumour-infiltrating lymphocytes display higher Sphk1 expression as compared to splenocytes in B16-F10-bearing mice [164]. Melanoma antigen-specific T cells deficient for Sphk1 (pMel-SphK1^−/−^ T cells) were shown to maintain a central memory phenotype and have a reduced propensity to differentiate into Treg as compared to wild-type T cells (pMel T cells). Tumour growth was significantly slower upon adoptive transfer of pMel-SphK1^−/−^ T cells, as compared to mice injected with wild-type pMel T cells [164].

Recent findings also show that S1P secretion, via the S1P transporter Spinster Homologue 2 (Spns2), reduced CD8^+^ T cell function and therefore promoted lung metastasis. Indeed, the deletion of Spns2, either globally or in a lymphatic endothelial cell-specific manner, was associated with an increased ratio of effector T-cells to immunosuppressive Tregs, in the lungs of Spns2-deficient mice intravenously injected with B16F10 or HCmel12 murine melanoma cells. This resulted in a reduced pulmonary metastatic burden as compared to what was observed in wild-type animals [165].

### 5.3. Ceramide and Its Derivatives in the Immune Response

Several studies have shown that ceramide metabolism could regulate the immune response in different melanoma models. Firstly, A-SMase-deficient B16-F1 melanoma cells engrafted in mice display an inflammatory TME and are infiltrated by high levels MDSCs and Tregs and low levels of DCs. A-SMase overexpression in these cells restores CD8^+^ and CD4^+^ T cells and DCs infiltration while reducing levels of infiltrating MDSCs and Tregs, thereby reducing tumour growth [166].

Secondly, KRN7000, a synthetic alpha-galactosylceramide [167], showed promising results in enhancing NK, NKT, CD8^+^ T cells and M1 infiltration in the syngeneic murine B16 metastatic melanoma model [168] but further investigation needs to be conducted.

Thirdly, it was recently reported that liposomes enriched in C2-ceramide were shown to reprogram the immune TME in a PKCζ-dependent manner in B16-F10-bearing mice. Under these conditions, TAMs shifted towards an M1 phenotype and CD8^+^ and Th1 cells infiltration was enhanced while intra-tumour MDSCs and Tregs levels were reduced [169].

Finally, gangliosides also represent attractive targets for immunotherapies as they are abundant in melanoma cells [24] and recognised by NKT cells [170]. GM2 [171] and N-glycolyl GM3 (NGcGM3), in particular, have been the main gangliosides used as targets for the development of anti-melanoma antibodies and vaccine [172,173]. In addition, gangliosides are also known to be shed by melanoma cells [174,175] and exert a pro-apoptotic effect on DCs [176,177]. 3F8, a monoclonal anti-GD2 mAb, demonstrated anti-proliferative and pro-apoptotic activity in human melanoma cell lines [178] but clinical studies focused on neuroblastoma [179] and medulloblastoma [180] patients. More recently, GD2 has been considered as a promising target for the treatment of melanoma patients using either CAR-T cell therapy [181] or the immunocytokine hu14.18-IL2, an anti-GD2 humanised mAb linked to two molecules of IL-2 and administered to patients with recurrent resectable stage III or IV melanoma [182].

### 5.4. Melanoma-Derived Exosomes Are Vectors of Immunosuppression

As mentioned above, Trajkovic et al. showed that the production of ceramide by nSMase 2 was part of the mechanisms involved in exosome budding [134]. In addition to favouring progression and metastasis, melanoma-derived small extracellular vesicles, often defined as exosomes, also carry immunomodulatory molecules that impair anti-tumour immune responses. In vitro, exosomes released from B16F0 murine melanoma cells inhibited the proliferation of T cells by delivering PTPN11(SHP-2) mRNA and protein [183]. B16F10-derived exosomes can also activate the mitochondrial apoptotic pathway of CD4^+^ T cells in vitro and in vivo, thereby increasing tumour growth and reducing T cell infiltration [184]. The authors proposed that the miRNA cargo of exosomes (e.g., miR-690) inhibited the expression of anti-apoptotic proteins in CD4^+^ T cells. In addition, small EVs produced by A375 human melanoma cells were shown to be able to reduce MHC class I molecules to the cell surface of primary human monocytes and THP-1 cells and downregulate the expression of endogenous MHC class I and II molecules in DCs [185]. Exosomes from metastatic melanoma-derived cell lines inhibited TCR signalling and cytokines secretion in CD8^+^ T cells by transferring an array of miRNA cargo [186]. Moreover, tumour-derived exosomes harvested from melanoma patients’ plasma were shown to induce the apoptosis, inhibit proliferation and decrease the activation of CD8^+^ T cells. They were also able to downregulate NKG2D expression on NK cells [187]. In metastatic melanoma patients, circulating exosomal PD-L1 suppressed CD8^+^ T cell activity. The authors reported that the pre-treatment level of circulating exosomal PD-L1 was a better predictor of clinical response to anti-PD-1 therapy than total circulating PD-L1 [188]. Importantly, Poggio et al. showed that Pdl1 knockout or exosome depletion by knocking out Smpd3, the gene encoding nSMase2, was sufficient to restore the anti-tumour immune response and to induce an efficient anti-tumour immune-memory response in the murine TRAMP-C2 prostate cancer model [189].

The relationship between SL metabolism and exosome-mediated immunosuppression in melanoma is not well understood, yet it could be a major mechanism of resistance to immunotherapy and thus deserves further investigation.

## 6. Potential Therapeutic Strategies for Melanoma Patients

Historically, when tumour resection was not possible or failed, chemotherapy was used to treat melanoma. dacarbazine (DTIC) has been approved as first-line treatment for advanced-stage melanoma and has remained for more than 30 years the standard chemotherapy despite no clear overall survival benefits [190,191,192].

The identification of melanoma driven mutations such as BRAF V600E allowed for a real breakthrough in the treatment of patients with metastatic melanoma. The emergence of BRAF targeted agents such as vemurafenib [54] and dabrafenib [55] allowed tremendous progresses in the field of personalised medicine and demonstrated survival benefits in metastatic melanoma patients as compared to dacarbazine-treated patients. Subsequently, the MEK inhibitor was also approved as treatment for this pathology as it showed survival benefits for patients displaying the BRAF V600E mutation [193]. Combination therapy using BRAF and MEK inhibitors such as cobimetinib [56] is nowadays one of the first line treatment for patients with BRAF V600E metastatic melanoma. This treatment results in higher rates as well as extended duration of response and decreases the cutaneous toxicities observed with the BRAF inhibitor monotherapy. Unfortunately, such therapeutic approaches remain constrained by the inevitable emergence of resistance to single-pathway blockade [194].

Immune checkpoint blockade (ICB) was the first therapeutic strategy to provide sustained responses and survival for advanced melanoma patients, even after treatment discontinuation [195,196,197]. Administering monoclonal antibodies targeting the immune checkpoint PD-1, alone or in combination with anti-CTLA-4 blocking antibodies is, to date, the standard of care for advanced melanoma patients. Independently of BRAF mutation status, patients treated with the anti-PD-1 and anti-CTLA-4 combo achieve at five years a progression-free survival and an overall survival of 36% and 52%, respectively [198]. Unfortunately, half of the patients do not respond or develop early resistance to ICB and exhibit severe immune-related adverse events (IRAE) [199]. Although treatment discontinuation due to adverse events seems not to affect the outcome for patients treated with ICB combination therapy [198], IRAEs tend to be associated with a better outcome for patients treated with anti-PD1 monotherapy [200].

Targeted therapies and ICB have deeply changed therapeutic management of patients with metastatic melanoma but all these therapeutic approaches still need improvement. Understanding the mechanisms that underlie resistance to these treatments is of utmost importance to improve the outcome of melanoma patients.

Here, we review some studies, which identified alterations in the SL metabolism as a cause of melanoma resistance to treatment (Table 2) and other studies using SL-related molecules as monotherapy or combined therapy to fight melanoma (Table 3).

## 7. Conclusions

**Figure 4:**
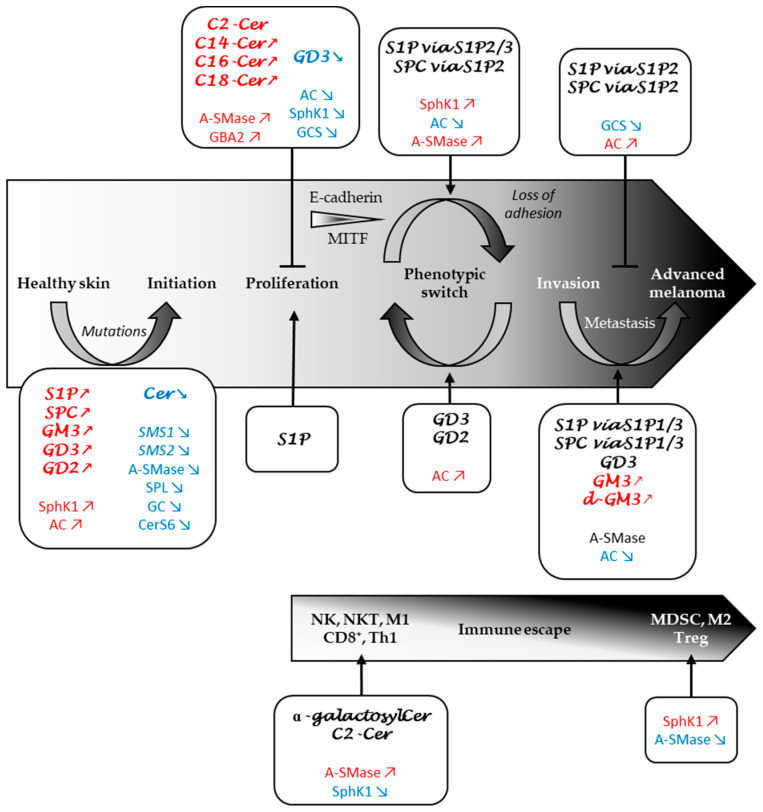
Role of sphingolipid metabolism in melanoma progression and immune response. SL metabolites and SL-metabolising enzymes whose levels and expression are increased, decreased or implicated are marked in red, blue or black, respectively. AC, acid ceramidase; CD8^+^, CD8^+^ T cells; Cer, ceramide; CerS, ceramide synthase; GC, glucosylceramidase; GCS, glucosylceramide synthase; M1, M1 macrophages; M2, M2 macrophages; MDSC, myeloid-derived suppressor cells; NK, natural killer cells; NKT, natural killer T cells; S1P, sphingosine 1-phosphate; S1P1/2/3, S1P receptor type 1/2/3; SMases, sphingomyelinases; SMS, sphingomyelin synthase; SPC, sphingosylphosphorylcholine; SphK, sphingosine kinase; SPL, S1P lyase; Th1, Th1 CD4^+^ T cells; Treg, tegulatory T cells.

As developed in this review, changes in SL metabolism that contribute to melanomagenesis, tumour progression and therapeutic resistance are multiple (Figure 4).

The action of sphingolipids (via the enzymes that control their metabolism, and transporters) is likely mediated by modifications in key regulatory processes including the phenotypic switch and EV-mediated cell-cell communication. Interestingly, these metabolic alterations could be envisioned as potential biomarkers and be exploited to better characterise tumour progression in melanoma patients. As a matter of fact, we identified that a reduced expression of SMS1 was significantly associated with a worse prognosis in metastatic melanoma [29]. AC was also identified as a potential biomarker for the prognosis of melanoma [211]. Moreover, we recently demonstrated that human invasive melanoma cells had lower AC levels and activity than proliferative melanoma cells [19]. In accordance, high AC expression was observed in node-negative stage II melanomas [18].

It is also interesting to note that a strong association between increased serum levels of gangliosides and high Breslow index or high Clark level as well as the presence of ulceration has been reported in melanoma patients, suggesting that circulating gangliosides may serve as potential markers for melanoma staging [212].

Monitoring the expression of SL-metabolising enzymes as well as SL levels could also be used to track the response to therapy in melanoma. Indeed, we previously showed that AC expression was associated to the response of melanoma cells to dacarbazine. Whereas overexpression of AC conferred resistance to dacarbazine, AC downregulation sensitised tumour cells to the drug [66]. DTIC triggered AC degradation and this effect was accompanied with an increased ceramide/S1P ratio. Our recent results also reveal that a distinct SL profile, i.e., a tendency for increased very long-chain ceramide species, was observed in the plasma of patients with melanoma who achieve a response to a BRAF-targeted therapy as compared with patients with progressive disease [64]. Finally, we recently discovered that melanoma patients with low SphK1 expression had significantly longer progression-free survival and overall survival than those with high SphK1 expression and patients with high SphK1 expression mostly failed to respond to anti-PD-1 therapy. These findings support the hypothesis that SphK1 expression represents a potential biomarker to predict tumour progression and resistance to anti-PD-1 in metastatic melanoma patients [22].

It would now be of great interest to evaluate the possible association between these SL metabolic alterations and the mutation status of oncogenes such as BRAF or NRAS as well as immune responses in metastatic melanoma patients. This will be performed in patients treated with anti-PD-1 in combination or not with anti-CTLA-4 in a prospective clinical trial (IMMUSPHINX: NCT03627026) we are currently conducting in our institute.

## Figures and Tables

**Table 1:** Impact of SL and SL-metabolising enzymes dysregulations on melanoma cell lines and patients.

SL or SL-Metabolising Enzymes	Dysregulation	Cell Lines or Patients	Effects	Refs
CerS6	Decreased	WM35, WM451 and SKMEL28 human melanoma cells	Malignant behaviour	[17]
AC	Decreased	Proliferative and invasive human melanoma cells	Pro-invasive	[18,19]
SphK1	Increased	Murine and human melanoma cells and biopsies	Pro-tumoral Immunosuppressive signature	[20,21,22]
SPL	Decreased	Human melanoma cells	Resistance to chemotherapy Increased proliferation	[23]
GD3	Increased	GD3+ human melanoma cells with c-Yes inhibition	Reduced malignancy	[24,25,26]
SMS1	Decreased	Human biopsies	Worse prognosis	[29]
SPC	Increased	Mel-Ab and human melanocytes	Stimulate melanomagenesis Hypopigmentation in melanocytes	[30,31,32,33]
A-SMase	Decreased	Primary melanomas and lymph node metastases Pigmented murine and human melanomas	Inverse correlation with melanin content	[34,35]

**Table 2:** SL-metabolising enzymes regulate the response of melanoma to therapy.

Targeted SL-Metabolising Enzyme	Melanoma Cells	Experimental Strategy	Treatment	Effects on Drug Sensitivity	Refs
A-SMase	B16-W6_pSIL10	shRNA	Cisplatin (chemotherapy)	Low A-SMase is associated with reduced mTOR-related autophagy and resistance to cisplatin	[201]
AC	A375	AC overexpression	Dacarbazine (chemotherapy)	AC overexpression confers resistance to dacarbazine	[66]
AC	G361 A375	ARN14988 ARN398 (AC inhibitor)	5-FU (chemotherapy)	AC inhibition sensitises G361 cells (proliferative phenotype) but not A375 cells (invasive phenotype) to chemotherapeutic drugs	[18]
SphK1	SK-Mel-28 A375	FTY720	Cisplatin (chemotherapy)	SK1 inhibition increases cisplatin-induced apoptosis through a downregulation of the PI3K/AKT/mTOR pathway and decreases EGFR expression	[202]
SphK1	UACC 903	siRNA	Staurosporine (Apoptosis inducing agent)	Downregulation of Sphk1 sensitises cells to staurosporine-induced apoptosis through AKT inhibition, and G0/G1 phase cell cycle arrest	[20]
SphK1	A375 (overexpression) Mel-2a (downregulation)	SphK1 overexpression or downregulation	Doxorubicin (chemotherapy)	Sphk1 overexpression induces resistance to doxorubicin-induced apoptosis whereas its downregulation by siRNA increases melanoma cell sensitivity to the treatment	[203]
SphK1	WM115 SK-Mel-28	FTY720	Vemurafenib (BRAF inhibitor)	SK1 inhibition increases vemurafenib-induced apoptosis	[204]
SphK1	WM9	SKI-I	Vemurafenib (BRAF inhibitor)	Sphk1 inhibition blocks BRAFi-resistant melanoma cell growth by reducing MITF and Bcl-2 expression	[64]
SphK1	B16-F10	PF-543	ICB Adoptive transfer of melanoma antigen-specific T cells	Sphk1 inhibition in T cells maintains Tcm phenotype, reduces Treg induction and synergises with anti-PD1 treatment	[164]
SphK1	Yumm 1.7	shRNA	ICB	SphK1 downregulation enhances ICB therapy efficacy by reducing Treg infiltration	[22]
GCS	B16	PDMP	Genistein (Apoptosis inducing agent)	Ceramide accumulation enhances genistein-induced apoptosis and growth inhibition through JNK activation and AKT inhibition	[205]

**Table 3:** SL-related molecules used therapy in melanoma.

SL-Related Treatment	Models	Associated Drug	Effects	Refs
Nanoliposomal ceramide	UACC 903 cells 1205 Lu cells Xenografts in nude mice	Sorafenib	Inhibition of melanoma cell growth by targeting both PI3K and MAPK signalling	[206]
Nanoliposomal ceramide	1205 Lu cells In vitro experiments	None	Reduction of integrin affinity and inhibition of melanoma cell migration through PI3K and PKCζ tumour-suppressive activities	[207]
KRN7000	B16 melanoma cell graft intravenously injected in mice	None	Increase of lifespan of mice	[208]
OGT2378 (GCS inhibitor)	B16 derived MEB4 melanoma cell graft in female C57BL/6 mice	None	Inhibition of tumour growth and reduction of established tumours	[69]
Intra-muscular GM3/VSSP vaccine	Phase I clinical trial: 26 patients with advanced (stage III and IV) melanoma	Adjuvant Montanide Isa 51	GM3/VSSP vaccine induces anti-GM3 IgM response in 44% of patients. Serum reactivity against melanoma cells and tumour biopsies is reported	[209]
L612-HuMAb (Human monoclonal antibody that binds to GM3)	Phase I clinical trial: 9 patients with advanced (stage IV) melanoma	None	L612 HuMAb induces significant antitumour activity in melanoma patients	[210]
